# Tai Chi as an Alternative Exercise to Improve Physical Fitness for Children and Adolescents with Intellectual Disability

**DOI:** 10.3390/ijerph16071152

**Published:** 2019-03-30

**Authors:** Zhaowei Kong, Tat-Ming Sze, Jane Jie Yu, Paul D. Loprinzi, Tao Xiao, Albert S. Yeung, Chunxiao Li, Hua Zhang, Liye Zou

**Affiliations:** 1 Faculty of Education, University of Macau, Macao, China; zwkong@um.edu.mo (Z.K.); tmsze@um.edu.mo (T.-M.S.); frank2011macau@gmail.com (H.Z.); 2 Department of Sports Science and Physical Education, the Chinese University of Hong Kong, Shatin, Hong Kong, China; jieyu0203@gmail.com; 3 Department of Health, Exercise Science and Recreation Management, The University of Mississippi, University, MS 38677, USA; pdloprin@olemiss.edu; 4 College of Mathematics and Statistics, Shenzhen University, Shenzhen 518060, China; taoxiao@szu.edu.cn; 5 Depression Clinical and Research Program at the Massachusetts General Hospital, Harvard Medical School, Boston, MA 02115, USA; ayeung@mgh.harvard.edu; 6 Physical Education and Sports Science Academic Group, National Institute of Education, Nanyang Technological University, Singapore 637616, Singapore; cxlilee@gmail.com; 7 Lifestyle (Mind-Body Movement) Research Center, College of Sports Science, Shenzhen University, Shenzhen 518060, China

**Keywords:** mind–body movement, aerobic exercise, balance, BMI, coordination, flexibility, developmental disability

## Abstract

Objective: The purpose of this study was to investigate the effects of Tai Chi (TC) on anthropometric parameters and physical fitness among children and adolescents with intellectual disabilities (ID). Methods: Sixty-six Chinese individuals engaged in sport-related extracurricular activities (TC and aerobic exercise (AE)) as exercise interventions or arts/crafts activities as a control condition (CON). The experimental protocol consisted of a baseline assessment, a 12-week intervention period, and a post-intervention assessment. Results: Significant interaction effect was only observed in the performance of a 6-min walk test. After 12 weeks of intervention, the AE group had significant changes in body mass index (*p* = 0.006, *d* = 0.11), sit-ups (*p* = 0.030 and *d* = 0.57), and 6-min walk test (*p* = 0.005, *d* = 0.89). Significant increases in vertical jump (*p* = 0.048, *d* = 0.41), lower-limb coordination (*p* = 0.008, *d* = 0.53), and upper-limb coordination (*p* = 0.048, *d* = 0.36) were observed in the TC group. Furthermore, the TC group demonstrated significantly greater improvements on balance compared to the control group (*p* = 0.011). Conclusions: TC may improve leg power and coordination of both lower and upper limbs, while AE may be beneficial for body mass index, sit-ups and cardiorespiratory fitness.

## 1. Introduction

Regular physical activity (PA) is essential for health development of children and adolescents [1]. Strong and consistent evidence has demonstrated that regular PA participation is associated with a reduced risk of becoming overweight or obesity, and the reduced likelihood of cardiovascular diseases, high blood pressure, and other metabolic dysfunctions [2]. The World Health Organization has recommended that all children and adolescents, including those with disabilities, should accumulate at least 60 min spent in moderate-to-vigorous-intensity PA each day [3]. However, children and adolescents with disabilities hardly meet this PA guideline [4] and are at a much higher risk of obesity when compared to their counterparts without intellectual disabilities (ID) [5]. A recent study showed that children and adolescents with ID participate in substantially less PA than other disability groups [6] and, thus, should be a target population for PA and health promotion.

Physical inactivity of individuals with ID may, in part, be influenced by their poor physical fitness [7]. Children with ID usually have a lower level of physical fitness (e.g., aerobic endurance, strength) and a higher level of adiposity in comparison to the general population, and such situations may persist into adulthood without effective interventions or remediation [8,9]. In recent decades, exercise programs have received greater attention in improving physical fitness in individuals with ID, and the exercise training is varied in its form with aerobic training being the most popular [10]. A meta-analysis by Shin and Park [11] reported that exercise interventions had a positive effect on both health- and skill-related physical fitness (e.g., cardiovascular fitness, muscular endurance, and muscular strength) in this population. However, it is worth noting that previous studies targeted adults with ID rather than children or adolescents with ID.

In adolescents with ID, a recent meta-analysis showed that exercise training has a significant positive effect on several components of skill-related physical fitness, including agility, power, and coordination, but the existing forms of exercise or therapy are not effective in improving balance [12]. Jeng et al. [12] suggested the need to improve lower limb strength due to its close association with balance in adolescents with ID.

Tai Chi (TC) is widely acknowledged as a feasible activity to improve functional capability and health for people with varied health conditions, such as older adults, patients with chronic diseases, and children with ID [13,14,15,16,17]. Features of TC emphasize dynamic shifting of body weight at a slow pace (require strong lower-limb fitness), integrated with breathing control, body awareness, and mental focus [18,19,20]. TC practice has been reported to be effective in improving physical fitness in numerous studies [21,22,23], yet few of them were conducted among individuals with ID. Notably, only two studies have evaluated the effects of TC on physical fitness in individuals with ID [24,25]. Azadeh et al. [24] demonstrated feasibility and effectiveness of TC practice on balance in female adolescents with ID. Kaplan et al. [25] utilized a one-group test–retest experimental design and found that the 24-week TC program improved balance, upper extremity reach, and respiration among older adults with moderate to profound ID. However, these two studies are largely limited by their study design (e.g., lack an active control group) and a small number of physical fitness outcomes assessed.

Therefore, the present controlled trial, with an active control group, aimed to examine whether a school-based TC program is effective in improving both health- (flexibility, body mass index, and body fat) and skill- (balance, coordination, muscular strength, muscular endurance, and leg power) related physical fitness in children and adolescents with ID. The information gained in this study will aid the development and implementation of feasible exercise programs in enhancing physical fitness and health in children and adolescents with ID.

## 2. Methods

### 2.1. Study Participants

To recruit participants, the principal investigator of this research project made contact with a K-12 school administrator (two local special schools and one integrated school), and informed them about the purpose of this study, the procedures involved, and the benefits of participating in this exercise program. To be included in this study, participants had to meet the following inclusion criteria: (1) aged 10 to 18 years old and be able follow an exercise intervention independently; and (2) be diagnosed with ID, with IQ scores below 70. Individuals were excluded if they: (1) were diagnosed with Down Syndrome; (2) had attended any structured exercise programs in the past 6 months; and/or (3) were smokers, alcoholics, and/or on medications. The research proposal was reviewed and approved by the Research Committee of University (Project Identification Number: MYRG 089(Y1-L2)-FED11-KZW). Written informed consent was obtained from legal guardians of the participants and school administrators.

### 2.2. Experimental Design and Study Procedures

**Figure 1:**
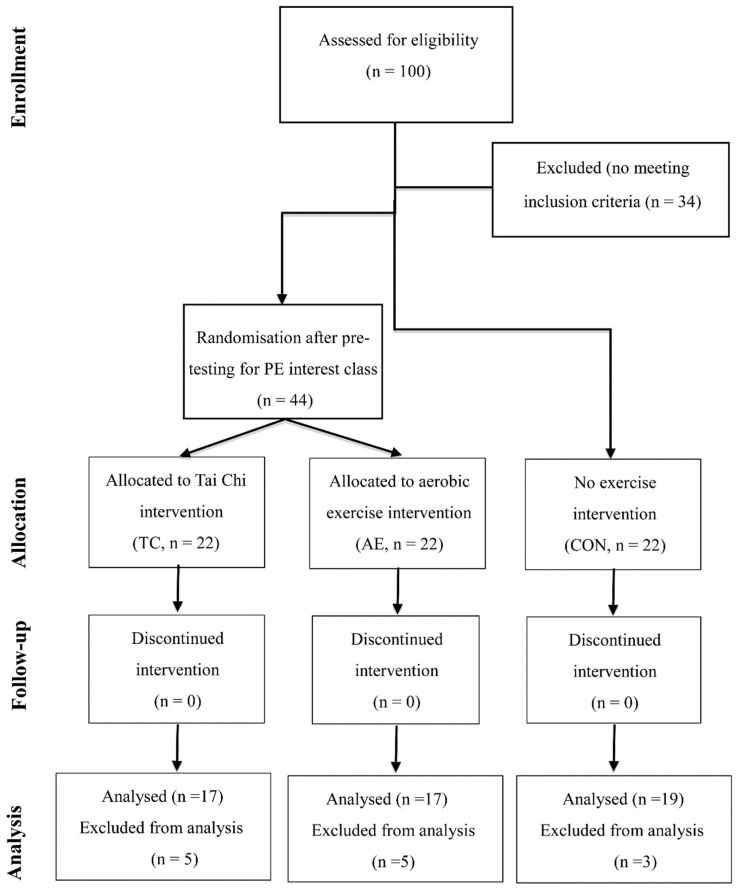
Flowchart shows study procedures including participant selection, outcome assessment, intervention protocol, and data analysis (PE = physical education, TC = Tai Chi, AE = aerobic exercise, CON = control group, PA = physical activity).

The experimental protocol consisted of a baseline assessment, a 12-week intervention, and a post-intervention assessment. Baseline- and post- intervention assessments were completed within one week prior to the intervention and following the last training session, respectively. Of note, all eligible participants attended two regular school physical education classes (sack race, parachute game, scooter board, tap shoulders, etc.) per week throughout the 12-week intervention period, with each class lasting for 40 min. Of the 100 Chinese individuals with ID that were screened, 34 were excluded because they did not meet the predetermined inclusion criteria. After the screening, 66 individuals volunteered to select either sport-related extracurricular activities (TC and aerobic exercise (AE)) as exercise interventions or arts/crafts activities as a control condition (CON). These extracurricular activities took place at the same time (14:00 to 16:00) as a school day. Furthermore, those individuals who selected sport-related activities were randomly assigned into either TC or AE training. Individuals who chose not to attend extracurricular activities (TC = 4, AE = 3, and CON = 3) and had an attendance rate of ≤60% (TC = 1, AE = 2), were excluded from the data analysis. The study procedures are presented in Figure 1.

### 2.3. Intervention Protocol

Participants in both TC and AE groups performed two 60-min sessions per week for 12 weeks in the indoor sports hall, while individuals in the CON group attended to a program involving arts and crafts activities in a usual classroom. All the TC training sessions were administered by a TC master with more than 10 years of teaching experience. In each TC training session, the participants started with a 10-min warm-up (jogging and muscular stretching), followed by 40-min TC practice and a 10-min cool-down. Given that the participants had cognitive impairments, a customized 8-form TC routine (without including beginning and closing movements) was developed by the TC master, including (1) Reverse Reeling Forearm; (2) Brush Knee and Step Forward; (3) Parting the Wild Horse’s Mane; (4) Cloud Hands; (5) Golden Rooster Stands on One Leg; (6) Left and Right Heel Kick; (7) Ward Off, Rollback, Press and Push; and (8) Cross Hands. Participants in the AE group underwent two 60-min aerobic dance sessions per week (i.e., 10-min warm-up activities, 40-min aerobic dance workout, and 10-min cool-down activities). This AE program was administered by a certified physical educator and personal trainer. Additionally, all participants were asked to maintain their normal daily activities including two regular physical education classes and restrain from extra exercises throughout the 12-week intervention. Exercise intensity during the exercise interventions were recorded by a heart rate (HR) system (Zephyr BioHarness, Auckland, New Zealand) during the entire period at three time points (week 4, week 8, and week 12), while steps were monitored using a pedometer (Ymax SW-200 digiwalker, Yamax Corporation, Tokyo, Japan) at four time points (week 3, week 6, week 9, and week 12).

### 2.4. Outcome Assessment

#### 2.4.1. Anthropometric Assessment

Anthropometric parameters, including body mass, standing height, skinfold (subscapular, calf, triceps) thickness, and waist and hip girths, were measured. Standing height in bare feet was measured and recorded as the nearest 0.1 cm. Body mass was assessed using the Bioelectrical Impedance Analyser (Tanita MC-180M, Tanita Corporation, Tokyo, Japan) in light clothing and recorded to the nearest 0.1 kg. Body mass index (BMI in kg·m^−2^) was computed by dividing weight (kg) by squared height (m^2^). Waist circumference was measured at the level of the smallest circumference above the umbilicus and below the xiphoid appendix. The subcutaneous skinfolds at three sites (triceps, subscapular, and calf) were made on the right side of the body using a Harpenden caliper (British Indicators, Hertfordshire, UK). If the difference between duplicate measures exceeded 1 mm for skinfolds or 1 cm for the girths, a third measurement was taken. The mean of the two closest duplicate or median of triplicate anthropometric measurements was used in the analysis. Intra-class correlation coefficients were between 0.89 to 0.99 for anthropometric measures of girth and skinfold.

#### 2.4.2. Components of Physical Fitness

Components of physical fitness consisted of flexibility, balance, coordination in upper and lower extremities, muscular strength (grip strength), leg power, muscular endurance, and cardiorespiratory fitness. Flexibility was measured using the Sit and Reach Test [26], where participants were instructed to push the sliding metal forward as far as possible; distance between the starting point and the place where the sliding metal stopped was recorded, with a longer distance indicating greater flexibility. Balance was measured using the Single-Leg Standing Test [27], where participants were asked to stand with one supporting leg while another leg was bent at roughly 90 degree, with a longer duration indicating greater balance performance. The Hopscotch Test [28] and the Turn-Over-Jars Test [29] were used to evaluate eye-foot and eye-hand coordination, respectively. Grip strength [30] was measured with a digital dynamometer (TKK 5401, Takei Scientific, Niigata, Japan). Lower-limb leg power was measured by the Vertical Jump Test [31], with a jump meter (TKK 5106, Takei Scientific, Niigata, Japan). Three trials for the above tests were performed and the best performance was considered for data analysis. The One-Minute Sit-up Test and the One-Minute Push-up Test were used to measure muscular endurance. Intra-class correlation coefficients ranged from 0.84 to 0.98 for these physical fitness measures. The 6-min walk test (6MWT) is a simple test to assess cardiorespiratory fitness [32]. The 6MWT was performed over a 200-m-long sports field, which was marked with a cone at every 10 m and a colored tape every 5 m. The number of meters walked was recorded.

### 2.5. Statistical Analysis

Statistical analyses were performed using the PASW software (Release 22.0; IBM, New York, NY, USA). Chi-square tests and one-way Analysis of Variance (ANOVA) tests were conducted for categorical data and continuous baseline data, respectively. Given that physical fitness classification is a common way to demonstrate an individual’s fitness level regardless of age and sex, we categorized the continuous variables of the observed physical fitness values into ordinal scale outcomes [33], which included five ranks according to local norms [34]. Before the main statistical analyses, the Shapiro–Wilk test was conducted in the outcome variables to verify the normality assumption. The main effects (time or group) and interaction effects (time × group) on the outcome variables were determined using two-way mixed ANOVA with repeated measures. Simple effect tests (Tukey) were performed if there was a time, group, or interaction effect. Partial eta squared (*η^2^*) was used as effect size estimates to measure of the main and interaction effects, which were considered as ‘small’ when *η^2^* = 0.01, ‘medium’ when *η^2^* = 0.09, and ‘large’ when *η^2^* = 0.25 [34]. Cohen’s *d* was used to reflect the magnitude of the intervention effect, and *d* = 0.2 is considered a ’small’ effect, 0.5 represents a ‘medium’ effect and 0.8 denotes a ‘large’ effect [35]. Results were presented as mean ± standard deviation (M ± SD), and *p* < 0.05 was considered as a statistically significant difference.

## 3. Results

No significant group differences at baseline were found in terms of ID, age, height, and weight, indicating that three groups had similar features (Table 1).

During the exercise intervention, participants in the TC group (HR mean: 97 ± 9 bpm at week 4 and 98 ± 6 bpm at week 8) had lower exercise intensities than the AE group (HR mean: 105 ± 8 bpm at week 4 and 109 ± 8 bpm at week 8) (all *p* < 0.05). There were no significant group differences in steps at all the four measurement time points, as well as in HR at week 12 (all *p* > 0.05).

### Anthropometric Parameters and Physical Fitness

Results of the primary outcomes are presented in Table 2 and Table 3. Anthropometric parameters and physical fitness characteristics (except for upper-limb coordination) at baseline were not significantly different among the three groups.

Significant interaction effects were only found in 6MWT (*p* = 0.034, *η^2^* = 0.129). In addition, we found a significant group effect on balance (*p* = 0.039, *η^2^* = 0.122), in which the TC group demonstrated a significantly greater improvement on balance as compared to the control group (*p* = 0.011), and from its own baseline (*p* = 0.332, *d* = 0.21).

There were main time effects, with medium to large effect sizes, in BMI (*p* = 0.006, *η^2^* = 0.143), sit-ups (*p* = 0.008, *η^2^* = 0.131), vertical jump (*p* = 0.019, *η^2^* = 0.106), upper-limb coordination (*p* < 0.001, *η^2^* = 0.300), lower-limb coordination (*p* < 0.001, *η^2^* = 0.244), and cardiorespiratory fitness (*p* < 0.001, *η^2^* = 0.210). Further analyses indicated that the AE group had a significant improvement in BMI (*p* = 0.006, *d* = 0.11), sit-ups (*p* = 0.030 and *d* = 0.57), and 6MWT (*p* = 0.005, *d* = 0.89). In addition, main time effects, with large effect sizes, were found in upper-limb coordination (*p* < 0.001, *η^2^* = 0.300) and lower-limb coordination (*p* < 0.001, *η^2^* = 0.244). Additional analyses demonstrated significant and moderate improvement in vertical jump (*p* = 0.048, *d* = 0.41), lower-limb coordination (*p* = 0.008, *d* = 0.53), and upper-limb coordination in the TC group (*p* = 0.048, *d* = 0.36). 

## 4. Discussion

The results of our controlled trial showed that both TC and AE programs are effective in improving physical fitness among children and adolescents with ID. Given that the 8-form TC is easy to master and practice, we recommend the simplified TC program for this special population. Our study findings are further discussed below.

### 4.1. Tai Chi Versus Aerobic Exercise Program in Exercise Intensity

It is widely accepted that TC, in general, is an aerobic exercise with low to moderate levels of intensity [36,37,38]. To determine exercise intensity of the 8-form TC, we measured participants’ HR responses during practice at week 4, week 8, and week 12. As our participants’ age ranged between 10 and 18 years, their maximum HR of 202 to 210 bpm was obtained through subtracting their age range from 220 [39,40]. The HR of participants in the TC group were between 97 and 101 bpm at the three measurement points (i.e., 101 to 147 bpm = 50% to 70% of the maximum HR), suggesting the 8-form TC is a program with low to moderate exercise intensity. In general, the TC group showed significantly lower HR response than the AE group. This result suggests that the 8-form TC training may cause less physical fatigue in this special population and may subsequently increase their exercise adherence. Step counting has been considered as a popular method of measuring distance [41]. As expected, no significant differences on this outcome was observed between the TC group and the AE group. Therefore, these two programs may induce similar benefits to the participants’ cardiorespiratory fitness. Heart rates during training at week 4, 8, and 12 are presented as supplementary data (Table S1: Training load during intervention).

### 4.2. Anthropometric Parameters

Accumulating evidence has shown that children and adolescents with ID are less physically active than their typically developing peers, which could put this special population at greater risk of developing obesity [42,43]. Such physical inactivity may be due to inadequate social and financial support and opportunities, problems with transport, the nature of multidimensional impairments, and lack of information on appropriate exercise modalities [44,45]. Thus, more effective exercise training options that are particularly suitable for this special group should be further explored. In the present study, we explored the effectiveness of the TC program that was specifically designed for children and adolescents with ID. To determine its effects on obesity related parameters, we collected participants’ BMI, waist to hip ratio, and skinfolds. It is well-known that in clinical practice and epidemiological studies, BMI is the most commonly used measure to classify overweight and obesity [46]. Interestingly, we observed a significant increase in BMI (*d* = 0.11) in the CON group. This increase may be due to normal growth and maturation [47]. In addition, we did not observe any training effect on waist to hip ratio and skinfolds. These insignificant findings on parameters related to obesity may be explained by a floor effect, as the participants were within a normal weight range. Thus, it awaits further investigations on whether TC training is effective on body composition in overweight and obese individuals with ID.

### 4.3. Selected Components of Physical Fitness

Children [8,48] and adolescents [49] with ID have demonstrated lower levels of physical fitness in comparison to their counterparts without disabilities. Previous studies had shown that individuals with ID had lower performance on muscular strength, trunk flexibility, muscular endurance, balance, leg power, and motor coordination [50,51,52,53]. This poor performance may lead to greater difficulty performing fundamental movement skills, potentially resulting in lower quality of life [43]. Physical exercise is widely accepted as a useful strategy to maintain and enhance physical fitness. In the present study, sit-ups in the AE group and four physical fitness outcomes (balance, leg power, upper-limb coordination, and lower-limb coordination) in the TC group were significantly improved after the exercise intervention. These positive findings parallel early review studies [12,54,55] synthesizing existing evidence regarding the effects of physical exercise training on physical fitness levels among individuals without ID. Our results also extended these early studies [25,26], in that TC practice can not only improve balance but other fitness outcomes among people with ID. 

### 4.4. Strength and Limitations of the Present Study

Strengths of this study include the use of standardized tests, predetermined eligibility criteria, randomized allocation of two experimental groups, qualified TC and AE instructors, and the customized 8-form TC. However, this study is not without its limitations. Firstly, this was not a randomized controlled trial because eligible participants with ID were allowed to select either sport-related programs or arts/crafts courses according to their own interests. Individuals who selected sport-related programs may have a greater expectation regarding the beneficial effects of exercise participation. Secondly, one of the investigators was the lead assessor who was not blinded to all experimental procedures, which may bias the assessment results. Thirdly, an improved trend on some physical fitness outcomes (e.g., grip strength and push-ups) were observed but did not reach statistical significance, which may be due to the relatively small sample size. The effect sizes obtained in the present study can be used for sample size calculations in future trials. Finally, because of the restriction of school curriculum, all participants received regular physical education classes while receiving exercise interventions. We are therefore unsure if the positive findings in two exercise groups are attributed to exercise training alone or from integrated effects (TC or AE training plus physical education).

## 5. Conclusions

The results of the present study indicate that two different training programs have unique benefits on measures of physical fitness. Furthermore, TC may improve leg power and coordination of both lower and upper limbs, while AE may be beneficial for body mass index, sit-ups, and cardiorespiratory fitness. In addition, it seems that the 8-form TC is superior to the AE program on improving balance. However, these results should be interpreted cautiously based on the stated limitations of this study. More rigorous studies with a larger sample size should be conducted to further confirm the results of this study.

## Figures and Tables

**Table 1:** Baseline parameters for participants.

Variable	TC (15M/2F)		AE (14M/3F)		CON (16M/3F)	
ID level						
I (50 to 69)	10	(58.8)	8	(47.1)	11	(57.9)
II (35 to 49)	5	(29.4)	6	(35.3)	5	(26.3)
III (20 to 34)	1	(5.9)	3	(17.6)	3	(15.8)
IV (<20)	1	(5.9)	0		0	
Age (years)	15.0	± 1.8	14.8	± 2.1	14.8	± 1.8
Height (cm)	160.9	± 13.3	157.8	± 11.9	161.3	± 14.0
Weight (kg)	53.6	± 20.1	53.2	± 15.1	56.9	± 18.5

TC, Tai Chi group; AE, aerobic exercise group; CON, control group; M, male; F, female; ID, intellectual disability; I, mild ID; II, moderate ID; III, severe ID; IV, profound ID.

**Table 2:** Anthropometric and physical fitness outcomes before and after 12 weeks of intervention.

Variable	TC (*n* = 17)				AE (*n* = 17)				CON (*n* = 19)				Time effect			Group effect			Interaction effect		
		Pre		Post		Pre		Post		Pre		Post									
	M	± SD	M	± SD	M	± SD	M	± SD	M	± SD	M	± SD	*F*	*p*	*η^2^*	*F*	*p*	*η^2^*	*F*	*p*	*η^2^*
BMI (kg⋅m-^2^)	20.2	± 5.1	20.4	± 4.8	21.0	± 4.2	21.5	± 4.2 ^a**^	21.5	± 5.28	21.7	± 5.0	8.323	0.006	0.143	0.350	0.706	0.014	0.526	0.594	0.021
W-girth (mm)	72.6	± 14.0	71.8	± 13.2	74.1	± 3.2	73.6	± 3.3	75.3	± 3.0	74.3	± 3.1	3.510	0.067	0.066	0.178	0.837	0.007	0.168	0.846	0.007
H-girth (mm)	85.3	±12.4	84.3	± 12.2	86.3	± 10.1	86.6	± 10.2	88.6	± 12.6	88.8	± 11.6	0.421	0.519	0.008	0.517	0.600	0.020	2.385	0.102	0.087
WHR	0.85	± 0.05	0.85	± 0.05	0.84	± 0.06	0.85	± 0.06	0.85	± 0.06	0.83	± 0.10	0.254	0.616	0.005	0.138	0.872	0.005	1.059	0.354	0.041
ΣSKF (mm)	40.7	± 26.9	38.7	± 22.7	40.5	± 17.3	39.9	± 18.0	45.7	± 23.6	44.6	± 23.6	3.099	0.084	0.058	0.336	0.716	0.013	0.348	0.708	0.014
GS	2.9	± 1.3	3.1	± 1.3	2.4	± 1.3	2.5	± 1.50	2.4	± 1.0	2.4	± 1.0	0.134	0.716	0.003	1.386	0.260	0.053	0.202	0.818	0.008
Sit-ups	1.1	± 0.2	1.2	± 0.4	1.2	± 0.5	1.6	± 0.9 ^a*^	1.3	± 0.7	1.4	± 1.0	7.547	0.008	0.131	0.847	0.435	0.033	1.667	0.199	0.063
Push-ups	1.2	± 0.5	1.4	± 0.7	1.0	± 0.0	1.0	± 0.0	1.1	± 0.5	1.2	± 0.5	1.709	0.197	0.034	1.984	0.149	0.076	0.779	0.465	0.031
Sit & reach	2.5	± 1.1	2.6	± 1.0	2.6	± 0.7	2.7	± 0.7	2.2	± 1.2	2.5	± 1.2	2.462	0.123	0.047	0.205	0.815	0.008	0.296	0.745	0.012
Balance	1.7	± 1.0	1.9	± 1.2	1.5	± 0.8	1.5	± 0.7	1.3	± 0.5	1.2	± 0.4 ^b**^	0.172	0.680	0.003	3.470	0.039	0.122	0.933	0.400	0.036
VJ	2.5	± 1.3	3.0	± 1.3 ^a*^	2.3	± 1.3	2.6	± 1.5	2.2	± 1.2	2.3	± 1.3	5.836	0.019	0.106	0.743	0.481	0.029	1.302	0.281	0.050
Up-C (s)	15.3	± 5.3	13.5	± 4.9 ^a**^	18.6	± 5.2	17.3	± 6.0	15.4	± 4.9	13.4	± 3.7	21.000	<0.001	0.300	3.093	0.054	0.112	0.353	0.705	0.014
Low-C (s)	16.2	± 5.3	13.8	± 3.6 ^a**^	18.6	± 6.7	16.6	± 4.6	19.4	± 7.2	17.7	± 6.7 ^a*^	15.784	<0.001	0.244	1.942	0.154	0.073	0.131	0.878	0.005
6MWT (m)	626	± 136	671	± 171	615	± 151	756	± 156 ^a*^	673	± 161	694	± 103	13.002	<0.001	0.210	0.394	0.677	0.016	3.626	0.034	0.129

TC, Tai Chi group; AE, aerobic exercise group; CON, control group; The physical fitness values were transformed into the ranks of physical fitness according to the norms of Macau residents based on different ages and genders; Rank 1 stands for “poor”, Rank 2 for “fair”, Rank 3 for “average”, Rank 4 for “good” and Rank 5 for “excellent”. BMI, body mass index; W-girth, waist girth; H-girth, hip girth; WHR, waist hip ratio; ΣSKF; sum of the skinfolds of three sites; GS, grip strength; VJ, vertical jump; Up-C, upper body coordination; Low-C, lower body coordination; 6MWT, six-minute walk test. ^a^, compared to pre-test with * (*p* < 0.05) and ** (*p* < 0.01); ^b^, compared to the TC group with * (*p* < 0.05) and ** (*p* < 0.01).

**Table 3:** Comparisons in the change ratio (Δ%) of outcomes before and after intervention.

Variable	TC (*n* = 17)				AE (*n* = 17)				CON (*n* = 19)			
	M	± SD	*d*	Effect size	M	± SD	*d*	Effect Size	M	± SD	*d*	Effect Size
BMI (kg⋅m^−2^)	1.5	± 4.4	0.04	small	2.2	± 2.8	0.11	small	1.5	± 4.4	0.04	small
W-girth (mm)	−0.9	± 4.5	0.06	small	−0.6	± 4.3	0.04	small	−1.4	± 3.6	0.07	small
Sit-up (reps)	11.8	± 33.2	0.36	small	38.2	± 69.7	0.57	medium	6.1	± 30.0	0.12	small
Balance (s)	29.9	± 69.5	0.21	small	10.8	± 60.1	0.12	small	−2.6	± 31.1	0.25	small
VJ (cm)	43.4	± 75.6	0.41	small	14.6	± 34.9	0.23	small	3.1	± 25.0	0.04	small
Up-C (s)	−11.2	± 15.9	0.36	small	−7.1	± 18.4	0.23	small	−11.5	± 12.6	0.47	medium
Low-C (s)	−12.3	± 15.4	0.53	medium	−6.9	± 21.9	0.35	small	−8.4	± 10.8	0.25	small
6MWT (m)	7.9	± 21.3	0.29	small	26.4	± 29.4	0.89	large	6.0	± 17.2	0.15	small

TC, Tai Chi group; AE, aerobic exercise group; CON, control group; reps = repetition; s = second; cm = centimeter; The physical fitness values were transformed into the ranks of physical fitness according to the norms of Macau residents based on the ranks with different ages and genders. BMI, body mass index; W-girth, waist girth; VJ, vertical jump; Up-C, upper body coordination; Low-C, lower body coordination; 6MWT, six-minute walk test.

## Supplementary Materials

The following are available online at https://www.mdpi.com/1660-4601/16/7/1152/s1, Table S1: Training load during intervention.
