# Supplementary material for: Tai Chi as an Alternative Exercise to Improve Physical Fitness for Children and Adolescents with Intellectual Disability
Source: Int J Environ Res Public Health. 2019 Mar 30;16(7):1152. doi: 10.3390/ijerph16071152 (PMC6479776; doi:10.3390/ijerph16071152)
Supplement: Supplementary file 1 [file ijerph-16-01152-s001.pdf]

Table S1. Training load during intervention.

**Training load**		**Tai Chi**	**Aerobic Exercise**
HRmean (bpm)			
	week 4	97 ± 9	105 ± 8 *
	week 8	98 ± 6	109 ± 8 *
	week 12	101 ± 14	107 ± 13

HRmean, mean heart rate during class; *: $p < 0.05$.
